# Evaluation of visitation policies and family information practices in pediatric intensive care units in Turkey: a national survey

**DOI:** 10.1007/s00431-026-07013-2

**Published:** 2026-05-07

**Authors:** Serhat Emeksiz, Bayram Bayramov, Alper Oglakcioglu, Serhan Ozcan, Tanıl Kendirli

**Affiliations:** 1 Division of Pediatric Intensive Care, Department of Pediatrics, Faculty of Medicine, Ankara Yıldırım Beyazıt University, Ankara, Turkey; 2 Faculty of Medicine, Department of Pediatrics, Division of Pediatric Critical Care, Ankara Yıldırım Beyazıt University, Ankara, Türkiye 06800; 3 Division of Pediatric Intensive Care, Department of Pediatrics, Faculty of Medicine, Ankara Bilkent City Hospital, Ankara, Turkey; 4 Division of Pediatric Intensive Care, Department of Pediatrics, Faculty of Medicine, University of Health Sciences, Ankara Bilkent City Hospital, Ankara, Turkey; 5 Division of Pediatric Intensive Care, Department of Pediatrics, Faculty of Medicine, Ankara University, Ankara, Turkey

**Keywords:** Pediatric intensive care units (PICUs), Visitation policies, Family information practices, Family-centered care

## Abstract

Family-centered care, including open visitation and effective communication, is increasingly recognized as essential in pediatric intensive care. Despite growing international evidence supporting open visitation policies and family involvement, significant variation exists in implementation across different healthcare systems and cultural contexts. This study aimed to evaluate the current visitation policies and family information practices in pediatric intensive care units (PICUs) in Turkey. A web-based survey was conducted among 59 PICUs across Turkey, focusing on ICU characteristics, visitation policies, and the provision of family information. The survey was distributed through the Turkish Pediatric Intensive Care Society network, and responses were obtained from one designated representative per unit. The majority of the units (78%) allowed a parent to remain at the bedside for 24 h for non-intubated patients, adopting family-centered care. However, 91.5% of the units had at least one restrictive visitation policy. Sibling visitation was prohibited in 67.8% of units. Visitation restrictions were eased under certain circumstances, such as after a diagnosis of brain death (83.1%) or in end-stage illness (76.3%). Family information was provided regularly by physicians in 57.6% of the units, and 49.2% had a dedicated family meeting room. Statistically significant differences were observed between isolation room-only units and mixed-room units regarding 24-h bedside family presence, visit duration, and the location of family information provision (*p* = 0.012, *p* = 0.031, *p* = 0.023, respectively). None of the units permitted family presence during invasive procedures or cardiopulmonary resuscitation. *Conclusion*: While a substantial proportion of Turkish PICUs allow 24-h family presence for non-intubated patients, restrictive policies remain common for visitors and siblings. The prohibition on family presence during procedures represents an important gap in the implementation of family-centered care. These findings highlight opportunities for policy development and staff education.

**Table:** 

**What is Known:** • *Restrictive visitation policies in pediatric ICUs increase family stress and may prolong patient recovery.* • *Family-centered care with 24-hour family presence improves patient and family outcomes.* • *Visitation and family information practices vary widely between countries and institutions.*
**What is New:** • *This is the first national survey evaluating visitation policies and family information practices in Turkish pediatric ICUs.* • *Most Turkish PICUs allow 24-hour family presence but maintain some visitation restrictions.* • *Significant differences exist between isolation-room-only and mixed-room units regarding visitation and family information provision.*

**What is Known:**

• *Restrictive visitation policies in pediatric ICUs increase family stress and may prolong patient recovery.*

• *Family-centered care with 24-hour family presence improves patient and family outcomes.*

• *Visitation and family information practices vary widely between countries and institutions.*

**What is New:**

• *This is the first national survey evaluating visitation policies and family information practices in Turkish pediatric ICUs.*

• *Most Turkish PICUs allow 24-hour family presence but maintain some visitation restrictions.*

• *Significant differences exist between isolation-room-only and mixed-room units regarding visitation and family information provision.*

## Introduction

Admission to a pediatric intensive care unit (PICU) is a significant source of psychological stress and anxiety for pediatric patients and their parents and primary caregivers [1, 2]. Primary caregivers (including parents, grandparents, aunts, uncles, and other legal guardians) play a crucial role in the emotional and psychological support of critically ill children. Thus, valuing the needs of patients and their relatives, showing empathy, and ensuring their comfort should be core principles of sensitivity for physicians, nurses, and other staff working in PICUs. Visitation policies in intensive care, a crucial component of this issue, have been debated for both pediatric and adult patients for several years [3]. An adult study showed that restrictive visitation policies (limitations on visiting hours, number of visitors, and duration of visits) prolong the patient’s recovery process, increase the incidence of delirium, and increase the family’s pain and stress [4]. A flexible visitation policy is subject to some restrictions but can be modified according to the needs, preferences, and specific circumstances of the patient and hospital [5]. Furthermore, flexible visiting hours reduce family members’ worry and anxiety, increase satisfaction, and contribute to patients’ recovery [6]. In recent years, a trend has been observed toward a family-centered intensive care model that allows families to be at the bedside 24 h a day, especially in pediatric patients [7, 8].

Family-centered care (FCC) is a healthcare approach that recognizes the family as an integral part of the patient’s care team [9, 10]. In pediatric intensive care settings, FCC encompasses not only visitation policies but also effective communication practices, family presence during procedures and rounds, parental participation in bedside care, and psychosocial support for families [7–9, 11]. The presence of parents at the bedside has been associated with numerous benefits, including reduced anxiety and pain in children, improved parental coping and satisfaction, and enhanced parent–child attachment during critical illness [1, 2, 12]. Effective information-sharing is a cornerstone of FCC. Regular, structured communication between healthcare teams and families reduces anxiety, improves satisfaction, and facilitates shared decision-making [13, 14]. Recommended practices include daily physician-family conferences, bedside rounds with family participation, and clear communication protocols during transitions of care. However, implementation of these practices varies widely across institutions and countries [13–15]. Although many studies on the organization of visitation policies have been conducted in Europe and the United States, no study has examined this subject in detail in PICUs in Turkey [3, 5–8]. Understanding current policies is essential for identifying gaps, benchmarking against international standards, and developing evidence-based recommendations tailored to the Turkish healthcare context. Thus, we conducted a national survey to assess the approach in tertiary pediatric ICUs in Turkey. This study aimed to identify current pediatric intensive care visitation policies and evaluate family information practices.

## Materials and methods

A web-based survey was conducted to investigate current visitation policies and family information in PICUs in Turkey. This study was conducted in accordance with the criteria of the Declaration of Helsinki. As the survey questions did not contain any patient information, patient consent was not required. Our study was approved by the Ankara Bilkent City Hospital Local Ethics Committee (decision number TABED/1/1658/2025).

### Survey Form

The survey was developed by the research team based on a literature review on visitation and family information practices in ICU settings. Response formats included multiple-choice, yes/no questions, and categorical options. The survey included 28 questions covering. The survey focused on four specific areas:

1. PICU characteristics (bed capacity, patient population, staffing),
2. visitation policies (hours, duration, visitor eligibility, sibling visitation),
3. family information practices (timing, methods, frequency), and
4. family presence during procedures.

### Participants and Implementation of the Survey

The survey was pilot-tested with five pediatric intensivists not included in the final sample to assess clarity and completeness. Minor wording modifications were made based on pilot feedback. The final survey was announced via email to the “Pediatric Intensive Care and Emergency” group, which included all intensive care specialists and faculty members in Turkey. To ensure institutional-level responses and avoid duplication, we explicitly requested that only one representative from each PICU complete the survey. A total of 59 PICUs responded out of 59 units contacted (100% response rate). We did not receive multiple responses from any single institution.” Consent to participate in the study was obtained in the first question of the survey from centers that agreed to complete it. We requested that respondents confirm their responses reflected official unit policies rather than personal opinions. All units were located in tertiary hospitals and admitted patients with medical, surgical, and cardiac conditions. Pediatric intensive care specialists staffed all units. Reminder emails were sent at 2 weeks and 4 weeks. After the study announcement, participation was open for 4 weeks. Respondent characteristics (years of experience, academic position) were collected but are not reported in this manuscript to maintain institutional anonymity. This study was reported in accordance with the Checklist for Reporting Results of Internet E-Surveys (CHERRIES) checklist for web-based surveys [16].

### Definitions

We evaluated the visitation policies of the participating units based on whether they applied restrictions based on the following five criteria: presence of a parent at the bedside 24 h a day, visiting hours, duration of visits, number of visitors, and whether visitors under 18 years of age were allowed. Hospitals with no restrictions were defined as having an open visitation policy, whereas those with restrictions were defined as having a restrictive visitation policy. We also inquired whether flexibility in ICU visitation policies was permitted under exceptional circumstances. Units that allowed family members to remain at the bedside 24 h a day were defined as units that adopted family-centered care.

Family information hours refer to designated time periods when physicians provide information to families about their child’s medical condition, treatment plan, and prognosis. These information hours typically occur at scheduled times and may be conducted in a designated area in person or via telephone.

'An open visitation policy' was defined as allowing at least one parent to remain at the bedside 24 h per day. ‘Restrictive visitation policy’ was defined as limiting parental or visitor presence to designated hours. These definitions specifically refer to routine visitation and do not include family presence during medical procedures, Cardiopulmonary Resuscitation (CPR), or invasive interventions, which were assessed separately.

Responding units were divided into two groups according to whether all PICU rooms were isolation rooms: Group A: all intensive care rooms were isolation rooms (*n* = 32), and Group B: intensive care rooms were not all isolation rooms (*n* = 27).

## Data management and analysis

Data were stored on a password-protected institutional server accessible only to the research team. Descriptive analyses were performed on the data collected. Descriptive statistics included frequencies and percentages for categorical variables. Categorical variables were expressed as percentages (%). Statistical Package for Social Sciences (SPSS) 25.0 (SPSS Inc., Chicago, IL) was used for data analysis. Categorical data were analyzed using Pearson’s chi-square test and Fisher’s exact test, and a p-value of < 0.05 was considered statistically significant. This was an exploratory survey, and p-values were not adjusted for multiple comparisons.

## Results

### Characteristics of the hospitals and PICUs

Fifty-nine PICUs responded to the survey. The response rate was 100%. Of the participating PICUs, 44.1% (*n* = 26) were from university hospitals, 27.1% (*n* = 16) from training and research hospitals, 25.4% (*n* = 15) from city hospitals, 1.7% (*n* = 1) from state hospitals, and 1.7% (*n* = 1) from private hospitals. All participating PICUs provided medical and postoperative care exclusively to pediatric patients. In 1.7% of the units (*n* = 1), there were fewer than six beds; in 35.6% (*n* = 21), there were 6–12 beds; in 47.4% (*n* = 28), there were 13–20 beds; and in 15.3% (*n* = 9), there were > 21 beds. In 39% of the responding units (*n* = 23), only one pediatric intensive care specialist was present. In 54.2% of the responding units (*n* = 32), all rooms served as isolation rooms (Table 1).

**Table 1:** Characteristics of participating pediatric intensive care units (*n* = 59)

Characteristic	*n* (%)
Hospital type	
University Hospital	26 (44.1)
Training and Research Hospital	16 (27.1)
City Hospital	15 (25.4)
State and Private Hospital	2 (3.4)
PICU bed capacity	
< 6 beds	1 (1.7)
6–12 beds	21 (35.6)
beds	28 (47.4)
> 21 beds	9 (15.3)
Patient population	
Medical, surgical, and cardiac	59 (100)
Are all rooms isolated?	
Yes	32 (54.2)
No	27 (45.8)

### Gowning procedures and hand washing

During patient visits, 66.1% of the units (*n* = 39) provided visitors with gowns only, 18.6% (*n* = 11) provided gowns and shoe covers, and 1.7% (*n* = 1) provided shoe covers only. Eight units (13.6%) did not provide any protective equipment to visitors, and 45 (76.3%) required mask wearing during patient visits. In all units, visitors were required to wash their hands upon entering and leaving the unit.

### Visitation policies, bedside family presence, and non-parental visitors

Forty-six units (78%) reported that for non-intubated patients, family members could remain at the bedside as caregivers for 24 h if the family wished, and 13 units (22%) reported that family members were not permitted to remain at the bedside as caregivers. Seven units (11.8%) allowed 24-h family presence, even for intubated patients. Of the centers that permitted bedside caregivers, 80% allowed either the mother or father, 10% allowed only the mother, and 10% allowed parents and other relatives (e.g., aunts) to act as caregivers. Statistically significant differences were detected between the two groups for the questions “Is 24-h bedside family presence permitted for non-intubated patients?” and “What is the duration of patient visits in the intensive care unit?”. In the group of all rooms that were isolated (Group A), the 24-h bedside family presence rate was significantly higher at 90.6% (*n* = 29/32) compared to 63.0% (*n* = 17/27) in Group B units (*p* = 0.012). The odds of allowing 24-h presence were 5.69 times higher in Group A units than in Group B units (OR: 5.69, 95% CI: 1.39–23.24). Regarding the duration of patients' visits, a significant difference was observed between the groups (*p* = 0.031). In Group B, the majority of units (70.4%) restricted visits to 15 min, whereas in Group A, this rate was substantially lower (37.5%), with a higher proportion allowing visits of 30 min or more. Responses to the other questions about patient visitation and the comparison between the two groups (how many times per day visits are allowed, on which days visits are allowed, and whether visitors under 18 years are permitted) are summarized in Table 2.

**Table 2:** Responses of participating units to the family information question

Family Information Practices	Total n: 59 (%)	Group A Units n: 32 (%)	Group B Units n: 27 (%)	
Who provides family information?				0.31
Attending physician and resident together	35 (59.3)	18 (56.3)	17 (63.0)	
Attending physician and fellow together	14 (23.7)	6 (18.8)	8 (29.6)	
Fellow	6 (10.2)	5 (15.6)	1 (3.7)	
Attending physician	4 (6.7)	3 (9.4)	1 (3.7)	
On which days is family information provided?				0.487
Every day	34 (57.6)	19 (59.4)	15 (55.6)	
Only weekdays	25 (42.4)	13 (40.6)	12 (44.4)	
Where is the family information given?				0.023**
Family meeting room	29 (49.2)	17 (53.1)	12 (44.4)	
Patient’s room	19 (32.2)	13 (40.6)	6 (22.2)	
In front of the ICU entrance	11 (18.6)	2 (6.3)	9 (33.4)	
At what time is the family information given?				0.125
Before 12:00	15 (25.4)	7 (21.9)	8 (29.6)	
12:00–13:30	11 (18.6)	9 (28.1)	2 (7.4)	
13:30–15:00	33 (56.0)	16 (50.0)	17 (63.0)	
Do you provide information to families by phone?				0.548
Yes	32 (54.2)	18 (56.2)	14 (51.9)	
No	27 (45.8)	14 (43.8)	13 (48.1)	
Is a security officer present during family information inside the ICU?				0.059
Yes	18 (30.5)	13 (40.6)	5 (18.5)	
No	41 (69.5)	19 (59.4)	22 (81.5)	

*P* < 0.05 significant

### Family information provision

In 29 ICUs (49.2%), information was provided to family members in a family meeting room. When this question was compared between the two groups, a statistically significant difference was observed (*p* = 0.023). The surveyed units were asked whether family members were provided information by telephone when they called the ICU. Twenty-seven units (45.8%) reported that they did not provide information by telephone, and 32 units (54.2%) reported that they did provide family information by telephone. When asked how they communicated with relatives of patients who did not speak Turkish, 42 (71.2%) units reported using translators employed by the hospital, and 17 (28.8%) reported using the Ministry of Health translation line. During family information hours, a security officer was present in 41 (69.5%) and absent in 18 (30.5%) units. Responses to questions regarding family information and comparisons between the groups are presented in Table 3.

**Table 3:** Responses from participating units to patient visitation policy questions

Patient Visitation Practices	Total (*n* = 59)	Group A Units (*n* = 32)	Group B Units (*n* = 27)	*P* value
Visitation policy				0.234
Open visitation	5 (8.5)	4 (12.5)	1 (3.7)	
Restricted visitation	54 (91.5)	28 (87.5)	26 (96.3)	
Is 24-h bedside family presence allowed for non-intubated patients?				0.012*
Yes	46 (78.0)	29 (90.6)	17 (63.0)	
No	13 (22.0)	3 (9.4)	10 (37.0)	
Who is allowed to visit the patient?				0.234
Parents	54 (91.5)	28 (87.5)	26 (96.3)	
Parents and other elder relatives	5 (8.5)	4 (12.5)	1 (3.7)	
How many times per day is visitation allowed?				0.217
Once a day	50 (84.7)	26 (81.2)	24 (88.8)	
Twice a day	4 (6.8)	2 (6.3)	2 (7.4)	
Families may visit anytime	5 (8.5)	4 (12.5)	1 (3.8)	
Which days is visitation allowed?				0.847
Every day	38 (64.4)	21 (65.6)	17 (63.0)	
Weekdays only	18 (30.5)	9 (28.1)	9 (33.3)	
Monday–Wednesday–Friday	3 (5.1)	2 (6.3)	1 (3.7)	
Are patients’ siblings under 18 years allowed to visit?				0.307
Yes	5 (8.5)	2 (6.3)	3 (11.1)	
Decided based on clinical condition	14 (23.7)	10 (31.3)	4 (14.8)	
No	40 (67.8)	20 (62.5)	20 (74.1)	
Duration of visitation allowed				0.031*
15 min	31 (52.5)	12 (37.5)	19 (70.4)	
30 min	19 (32.2)	12 (37.5)	7 (25.9)	
45 min	6 (10.2)	6 (18.8)	1 (0)	
> 1 h	3 (5.1)	2 (6.3)	1 (3.7)	

*P* < 0.05 significant

### Flexibility in visitation policies for special circumstances

Participants were asked whether they had made changes to family presence or visitation policies in exceptional circumstances (after a diagnosis of brain death and for end-of-life patients). In response to the question of whether they allowed flexibility in the number of visitors or visitation policies (e.g., allowing other relatives to view the patient) after a diagnosis of brain death, 49 units (83.1%) answered yes, and 10 units (16.9%) reported no change. In response to the question of whether they allowed flexibility in the number of visitors or visitation policy for end-of-life patients, 45 units (76.3%) answered yes, and 14 units (23.7%) reported no flexibility.

### Interventional procedures, cardiopulmonary resuscitation, and special situations

In response to separate questions asking whether family members were permitted to remain at the bedside during interventional procedures (central venous catheter insertion, endotracheal intubation) and cardiopulmonary resuscitation, all 59 units (100%) reported that they did not permit this.

## Discussion

This study is the first to evaluate the status of patient visitation policies and family information practices in PICUs in Turkey. Notably, 78% of the units reported adopting family-centered care and stated that, for a non-intubated patient, a parent (preferably the mother or father) could remain at the bedside for 24 h. Moreover, 91.5% of the units had adopted at least one restrictive visitation policy (limitations on visiting hours, visiting days, the number of family members permitted simultaneously, or acceptance of visitors under 18 years of age). Additionally, the units reported easing visitation restrictions under certain circumstances, such as after a diagnosis of brain death (83.1%) or in end-stage illness (76.3%). In interpreting our findings, it is important to note that we defined ‘family-centered care’ operationally as permitting 24-h bedside family presence for non-intubated patients. This represents one important dimension of FCC but does not capture all elements, including communication practices, family presence during procedures, or family participation in decision-making. Therefore, our finding that 78% of Turkish PICUs adopt family-centered care should be understood within this limited operational definition and not as comprehensive FCC implementation.

In recent years, family-centered care has been recommended in PICUs to enhance care quality and health outcomes for children and families [11, 17]. While there is consensus that family presence in ICUs improves care, clinical outcomes, and family well-being, policies vary by country and unit [3, 18–20]. An open visitation policy doesn't necessarily mean continuous family presence; one study noted 58% 24-h family presence in a unit with an open visitation policy [21]. Despite restrictive policies, 78% of units allowed 24-h bedside presence for parents, showing Turkey's adoption of family-centered care, recognizing family members as caregivers. This approach began in the US with initiatives like “Together for Better” and is now globally endorsed for adult ICU [22, 23]. Our survey showed the more restrictive policy for mechanically ventilated patients, with only 11.8% of units allowing 24-h family presence for intubated children. This distinction is not consistently reported in the international literature, limiting direct comparisons. However, this practice may reflect concerns about parental distress when witnessing invasive procedures or critical events. Evidence suggests that with adequate preparation and support, families can be present even during critical illness phases without adverse effects on care delivery [24]. Family-centered care in ICUs benefits patients and relatives, allowing 24-h bedside presence as circumstances allow. Restrictions may apply to intubated patients or non-parental visits. We acknowledge that our study predominantly refers to ‘parents,’ but we recognize that primary caregivers may include grandparents, aunts, uncles, or other legal guardians. Future research should explicitly include all types of primary caregivers to more accurately reflect the diversity of family structures.

Visitation policies and family information practices in ICUs are closely related to family satisfaction [25]. Waiting outside the ICU is challenging for families; waiting rooms are crucial for satisfaction [4]. Many countries have family waiting rooms near ICUs for comfort and privacy, with amenities such as Internet access, microwaves, and coffee machines recommended [26, 27]. A survey found that 95.4% of units had waiting rooms with facilities such as refrigerators and showers [28]. However, only 49.2% of units participating in our survey had family information rooms, and none had structured ICU waiting rooms. This need should be considered in future hospital designs.

Opinions vary on conducting patient visits in intensive care units [29–31]. In an ICU survey study including 170 hospitals, it was emphasised that 32% of the units did not restrict visiting hours, but most limited visitor age and number [32]. A review showed flexible visitation led to shorter ICU stays, less delirium, and higher family satisfaction [33]. A French survey of 29 units found support for open visitation [34]. While open visitation boosts family satisfaction, it doesn't benefit ICU staff; flexible hours increased staff burnout [31]. Although only five units (8.5%) in our study adopted an open visitation policy, the rate of family presence at the bedside was 78%. Several factors influence parental bedside presence and the visitation policies. Maintaining a focus on patient- and family-centered care and planning visitation practices that consider patient and family satisfaction are important responsibilities of hospital administration and unit physicians. Visitation policies should be reviewed in detail on each platform, and their pros and cons should be carefully discussed.

The American College of Critical Care Medicine has published guidelines recommending a 24-h open visitation policy for family members. These guidelines also discuss the importance and implementation considerations for sibling visits [7]. In a study from Italy evaluating visitation policies, which included participation from 30 pediatric intensive care units, 23% of the units reported having 24-h visitation policies, and 37% allowed child visitors (i.e., siblings) [35]. In a survey that evaluated sibling visits, 66% of French intensive care units reported permitting sibling visits [34]. In the present study, 67.8% of the units did not permit sibling visits, while 23.7% allowed them, depending on the patient’s clinical condition. More studies are needed in this population, particularly on long-term psychological effects.

The literature contains both positive and negative reports on the presence of family during procedures and interventions in the intensive care unit [36, 37]. Family presence during endotracheal intubation was not associated with lower first-attempt success rates, adverse intubation events, oxygen desaturation, or higher team stress levels [38]. In a survey conducted in a unit that supported an open visitation policy, 84% of participants reported that a family member was present in the room during invasive procedures (e.g., intubation, central catheter insertion), and family satisfaction was high [39]. Some studies predict that open visitation policies and family presence during procedures may create physical and psychological stress for intensive care staff and impede patient care [40]. In the present study, allowing family presence in the patient’s room during invasive procedures was not an accepted approach. We believe that further research is needed to identify the reasons for barriers to family presence during invasive procedures in PICUs in Turkey, to understand healthcare professionals' concerns, and to develop policy change strategies.

Providing information to families is crucial for maintaining patients' and relatives' trust in intensive care units. Families want to receive clear, consistent, and accurate information about their patients directly from the responsible clinical physician and to be informed about the course of the illness. Effective and timely information provision is a fundamental component of family-centered care. International guidelines recommend daily physician-family communication as a minimum standard, with additional updates as clinically indicated [7, 41]. Studies have reported that family members want to receive complete information at least once daily about the patient’s clinical status, disease course, and treatment [4, 34]. In the present study, we observed that 57.6% of units provided family information daily, with at least one physician present. This finding suggests that pediatric intensive care units in Turkey recognise the importance of providing family information and place a high value on this practice. We also observed a statistically significant difference between Groups A and B regarding where family information was provided (*p* = 0.023). We believe that improving the physical conditions of intensive care units and, where possible, establishing units where every room is an isolation room would allow family information provision to be conducted under better conditions. Also, the lack of standardized communication protocols in many Turkish PICUs represents an opportunity for quality improvement.

In the present study, the survey responses were provided on behalf of the units by the unit chief of the pediatric intensive care unit. Therefore, the responses may reflect the intent rather than the actual practice. We also did not examine important determinants of visitation policies, such as the type of intensive care unit (e.g., academic versus non-academic, public versus private), staff competence, staff attitudes toward visitation, or sociocultural characteristics of the population. Our study documented that no participating PICUs allowed family presence during invasive procedures or CPR, but we did not systematically assess the reasons for these prohibitions. We consider these issues to be limitations of the present study. Another limitation of our study was that it did not elicit or explore patient/caregiver attitudes or the actual experience of visitation. Our survey assessed policies and practices but did not measure outcomes. We did not collect data on patient clinical outcomes, family satisfaction, psychological well-being of parents, or staff perceptions and burnout. The strengths of our study include the large proportion of pediatric intensive care units in Turkey and the fact that this is the first study on this topic in Turkey.

## Conclusion

This national survey provides the first comprehensive evaluation of visitation and family information practices in Turkish PICUs. Although 24-h family presence for non-intubated patients is commonly permitted, restrictive policies remain prevalent for non-parental visitors and sibling visitation. The universal prohibition on family presence during invasive procedures and cardiopulmonary resuscitation represents a significant gap in the implementation of family-centered care in Turkey. Significant structural differences were observed between the two groups, particularly regarding 24-h family presence at the bedside, visit duration, and the location of family information provision. These findings highlight the need for standardized, evidence-based visitation and communication policies in Turkish PICUs. Follow-up studies in subsequent years should be planned to assess whether practices change over time.

## Data Availability

No datasets were generated or analysed during the current study.

## Abbreviations

CHERRIES: Checklist for Reporting Results of Internet E-Surveys
CPR: Cardiopulmonary Resuscitation
FCC: Family-centered care
PICU: Pediatric intensive care unit
ICU: Intensive care unit
